# Prognostic impact of CD73 expression and its relationship to PD-L1 in patients with radically treated pancreatic cancer

**DOI:** 10.1007/s00428-020-02888-4

**Published:** 2020-07-16

**Authors:** Kyösti Tahkola, Maarit Ahtiainen, Ilmo Kellokumpu, Jukka-Pekka Mecklin, Johanna Laukkarinen, Joni Laakkonen, Istvan Kenessey, Sirpa Jalkanen, Marko Salmi, Jan Böhm

**Affiliations:** 1 Department of Surgery, Central Finland Central Hospital, Keskussairaalantie 19, 40620 Jyväskylä, Finland; 2 Faculty of Medicine and Health Technology, Tampere University, Tampere, Finland; 3 Department of Education and Research, Central Finland Central Hospital, Jyväskylä, Finland; 4 Sport &Health Sciences, University of Jyväskylä, Jyväskylä, Finland; 5 Department of Gastroenterology and Alimentary Tract Surgery, Tampere University Hospital, Tampere, Finland; 6 MediCity Research Laboratory and Institute of Biomedicine, University of Turku, Turku, Finland; 7 Department of Pathology, Central Finland Central Hospital, Jyväskylä, Finland

**Keywords:** Pancreatic cancer, Microenvironment, CD73, PD-L1, Prognosis

## Abstract

Immune suppressing molecule CD73 is overexpressed in various cancers and associated with poor survival. Little is so far known about the predictive value of CD73 in pancreatic ductal adenocarcinoma (PDAC). The purpose of this study was to investigate the prognostic significance of CD73 in PDAC. The study material consisted of 110 radically treated patients for PDAC. Tissue microarray blocks were constructed and stained immunohistochemically using CD73 antibody. Staining intensity and numbers of stained tumour cells, inflammatory cells, stroma, and blood vessels were assessed. High-level CD73 expression in tumour cells was positively associated with PD-L1 expression, perineural invasion, and histopathological grade. CD73 positivity in tumour-infiltrating lymphocytes was significantly associated with lymph node metastasis. Lymphocytic CD73 positivity was also associated with staining positivity in both stroma and vascular structures. In addition, CD73 positivity in vascular structures and stroma were associated with each other. There were no significant associations between CD73 positive tumour cells and CD73 positivity in any other cell types. PD-L1 expression was associated with CD73 staining positivity in stroma (*p* = 0.007) and also with histopathological grade (*p* = 0.033) and T class (*p* = 0.016) of the primary tumour. CD73 positivity in tumour cells was significantly associated with poor disease-specific (*p* = 0.021) and overall survival (*p* = 0.016). In multivariate analysis, CD73 positivity in tumour cells was an independent negative prognostic factor together with histopathological grade, TNM stage, and low immune cell score. In conclusion, high CD73 expression in tumour cells is associated with poor survival in PDAC independently of the number of tumour-infiltrating lymphocytes or TNM stage.

## Introduction

Tumour microenvironment has been shown to impact on cancer progression [1–4]. Malignant tumours like pancreatic ductal adenocarcinoma (PDAC) are known to develop several mechanisms in order to suppress the host immune system [5, 6]. In line with others, our previous results have shown an association between the number of tumour-infiltrating lymphocytes (TILs) and survival in various cancer types ([7–9]).

PDAC is the seventh deadliest cancer worldwide [10]. Approximately 80% of patients have an unresectable tumour at the time of diagnosis due to advanced disease [11] and survival rates remain low even after attempted curative surgery [12]. Despite the promising results of immune-modulating agents in many other cancers, the results in PDAC have been disappointing.

CD73, also called ecto-5′-nucleotidase (NT5E), is one of the major nucleotide metabolizing enzymes having an essential role in sustaining immune homeostasis. It dephosphorylates adenosine monophosphate (AMP) to adenosine, which in turn activates specific G protein-coupled receptors (GPCR) and suppresses immune reaction. The apical distribution of CD73 in normal pancreatic duct epithelial cells has been shown to shift to a more diffuse distribution in PDAC [13]. This probably promotes cancer cell aggressiveness, angiogenesis and metastasis [14–16]. CD73 also has non-enzymatic functions in cells, and there is evidence suggesting that CD73 also promotes the proliferation and migration of cancer cells independently of its enzymatic activity [17].

**Fig. 1:**
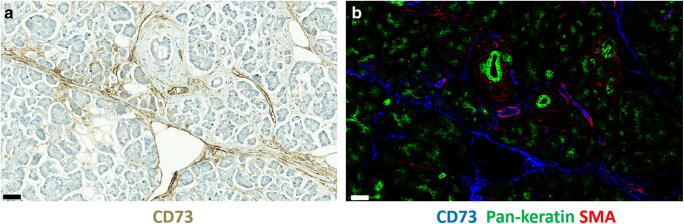
CD73 expression in normal pancreas. **a** Immunoperoxidase staining of normal pancreas for CD73 (brown). **b** Multicolour immunofluorescence staining of a consecutive section of normal pancreas for CD73 (blue), pan-cytokeratin (green) and alpha-smooth muscle actin (red). Bars, 50 μm

There are reports of CD73 overexpression in various cancers [18–24] showing an association with poor survival [16, 25]. However, opposite associations have also been reported [26]. One reason may be that CD73 is expressed in a variety of cell types such as certain lymphocyte populations, lymphatic and blood endothelial cells, subsets of epithelial cells, fibroblasts and cancer cells (Fig. 1**).** Often cell-specific expression has not been taken into account in these prognostic analyses. The prognostic value of CD73 in PDAC is still limited [27].

PD-L1 (also called B7-H1 or CD274) is an immunosuppressive molecule. According to earlier studies, high-level PD-L1 expression seems to be associated with poor differentiation, neural invasion and poor survival in PDAC [28].

Little is known so far about the prognostic impact of CD73 in PDAC, and there are no studies concerning co-expression and a possible interrelationship between CD73 and PD-L1. Targeting CD73 could be a novel cancer treatment strategy; it is currently under intensive research and several clinical trials are ongoing (www.clinicaltrials.gov).

The aim of this study was to ascertain whether cell-specific CD73 acts as a prognostic factor in PDAC and to evaluate its relationship to other factors in microenvironment, such as PD-L1 and immune cell score (ICS). This study was designed and performed according to the reporting recommendations for prognostic studies on tumour markers [29].

## Material and methods

From 2000 to 2016, a total of 110 patients with stage I-IV PDAC were operated on in the Central Hospital of Central Finland, Jyväskylä, Finland. The surgical procedures included 20 classic pancreaticoduodenectomies, 82 pylorus-preserving pancreatoduodenectomies, 4 total pancreatectomies, and 4 distal pancreatic resections. Data were retrieved from our prospectively maintained and continuously updated population-based database established in 2000, including detailed information on patient and tumour characteristics, surgical treatment and complications, oncological treatments and follow-up. Patients with tumour-node-metastasis (TNM) stage III-IV (*n* = 7) were excluded from the survival analysis. Neoadjuvant chemotherapy was not given to any of the patients, whereas 95% of patients received adjuvant chemotherapy.

### Histopathological examination

All histopathological tumour specimens were reviewed by an experienced gastrointestinal histopathologist (JB). Tumour staging was done according to the 7th edition of the UICC/AJCC TNM categories [30]. The grading was performed according to the WHO classification of tumours 2010 [31].

### Tumour sampling and immunohistochemistry

Tissue microarray blocks were constructed from formalin-fixed paraffin-embedded primary PDAC patient tumour samples. Two tissue cores 0.6 mm in diameter were taken both from the core of the tumour and the invasive margin from representative tumour blocks. Sections of 2 μm thickness were used for immunohistochemical (IHC) analyses. Staining for CD73 was conducted with rabbit monoclonal anti-CD73 antibody (D7F9A, Cell Signalling) and ultraView Universal DAB detection kit (Roche) for Ventana. Staining for CD3 and CD8 was conducted with anti-CD3 (LN 10, 1:200; Novocastra) and anti-CD8 (SP16, 1:400; Thermo Scientific) antibodies, using a Lab Vision Autostainer 480 (ImmunoVision Technologies Inc.). Staining for PD-L1 was conducted with anti-PD-L1 (E1L3N, 1:100; Cell Signalling Technology) antibody, using a BOND-III stainer (Leica Biosystems). PD-L1 staining was carried out using whole tissue sections.

Signal visualization for all IHC was done by diaminobenzidine and sections were counterstained with haematoxylin. In order to validate our TMA method for CD73, we analyzed the expression of CD73 in tumour cells (TC) using whole-section samples from 16 corresponding cases. The correspondence between whole sections and TMA punches was 100%; when assessing tumour cells separately in both groups, the same 4 samples out of 16 were considered CD73 positive in both groups.

ICS was determined using TMA technique as described earlier [7]. Briefly, ICS describes the immune response represented by CD3 and CD8 immune cells in the tumour centre and at the invasive margin.

For immunofluorescence stainings of FFPE samples, Alexa Fluor 488-conjugated anti-pan-cytokeratin (eBioscience #53-9003-80) and Cy3-conjugated anti-α-smooth muscle actin (Sigma #C6198 both mouse monoclonal antibodies) were used together with the rabbit anti-human CD73 antibody (D7F9A), which was visualized using Alexa647-conjugated goat anti-rabbit IgG (Invitrogen #A32733) as a second-stage reagent. The stained sections were imaged using Pannoramic Midi FL slide scanner (3DHISTECH) and analyzed using Case Viewer 1.4 program.

### Quantitative evaluation of CD73 and PD-L1 expression

**Fig. 2:**
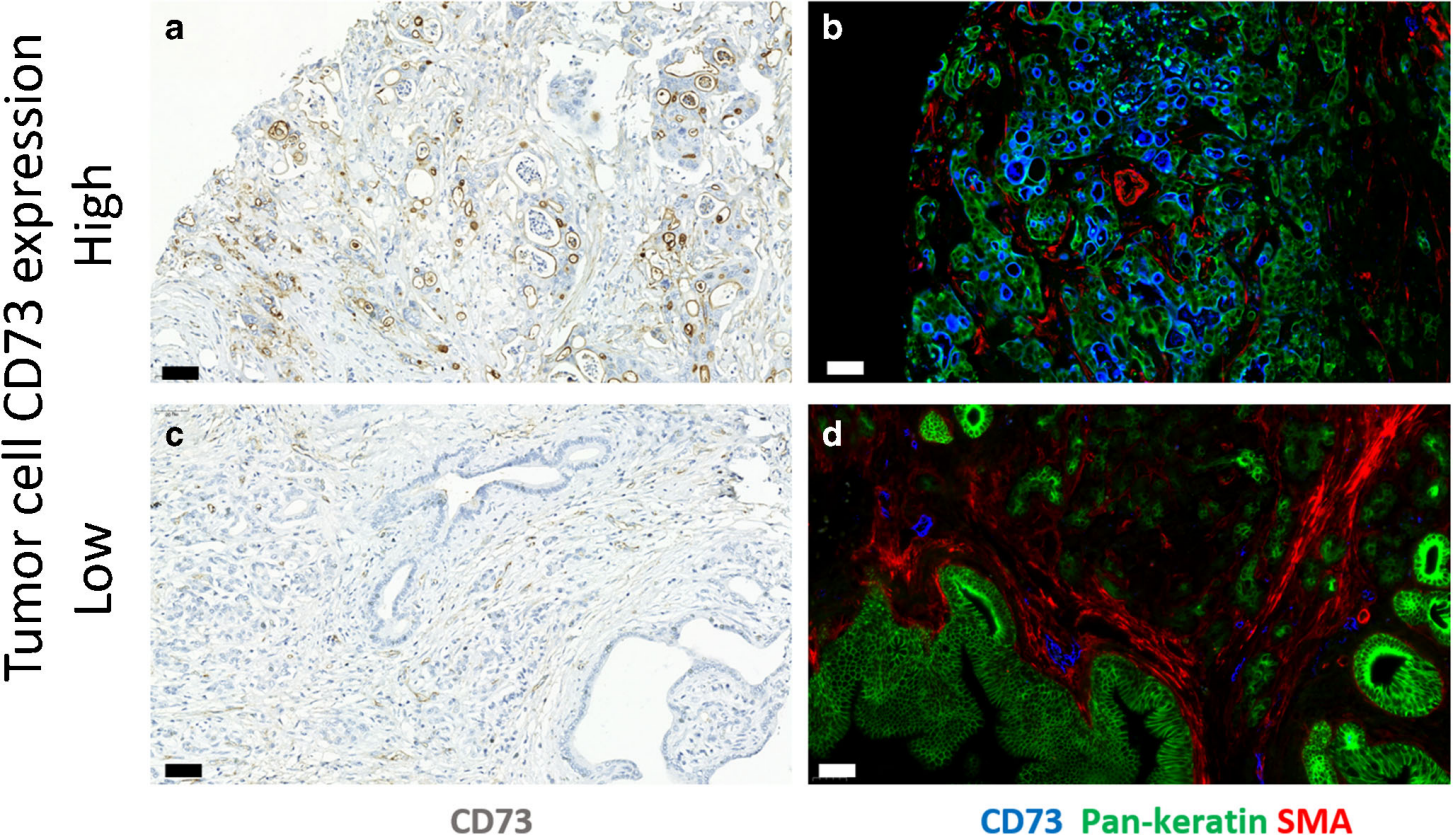
CD73 expression in adenocarcinoma of pancreas. Representative immunoperoxidase stainings (a and c) for CD73 (brown) and multicolour immunofluorescence stainings (b, d) for CD73 (blue), pan-cytokeratin (green) and alpha-smooth muscle actin (red). Bars, 50 μm

IHC-stained TMA slides were assessed independently by four researchers (IK, JL, KT, JB) blinded to the clinical data. In case of disagreement, consensus was reached by three observers. In the case of CD73 staining, the intensity (1–3) and the proportion of staining on the cell surfaces (0–100%) were assessed. The final score (0–300) was calculated by multiplying the proportion of stained tumour cells by the staining intensity. Patients were divided into two groups using a cut-off value of 90, which was selected by using receiver operating characteristic (ROC) curves drawn in relation to disease-specific 3-year mortality (Fig. 2**).**

In addition, the percentage of CD73 positive TILs, tumour stroma and vascular structures were assessed. In the case of TILs, the sample was considered positive if > 3% of lymphocytes were positive for CD73. Tumour stroma positivity was considered weak, moderate or strong when < 5%, 5–16% or > 17% of the stromal area was stained respectively. Due to the strong staining intensity of vascular structures, 95% was set as a cut-off value for CD73 positivity of vascular structures.

PD-L1 expression was evaluated by estimating the proportion of PD-L1 positivity on the tumour cell surface. If over 1% of the tumour cells expressed PD-L1, the tumour was considered positive. There is no consensus on how PD-L1 expression should be reported in PDAC, and therefore none of the schemes like tumour proportion scale (TPS) or combined positive score (CPS) was used.

### Statistical analyses

The associations between clinical and histopathological variables, cell-specific CD73 positivity and PD-L1 positivity in tumour cells were analyzed using chi-square test. Univariate and multivariate Cox proportional hazards regression model was used to calculate hazard ratios for OS and DSS. Only variables with *p* < 0.05 in univariate analysis were entered into the multivariate analysis despite the a priori determined confounder, tumour stage (*p* = 0.158). All statistical tests were two-sided. A *p* value less than 0.05 was considered significant. The statistical analyses were performed with IBM SPSS statistics 24 for Windows (IBM Corporation, Armonk, NY, USA).

## Results

### Patient demographics

A total of 110 of PDAC patients were included in this study. The distribution of samples regarding different variables is shown in Table 1.

**Table 1:** Clinicopathological characteristics

*n* = 110		
	n/median	%/min-max
Age, years	67.0	45–82
Gender		
Male	58	52.7
Female	52	47.3
T-stage		
pT1	3	2.7
pT2	22	20.0
pT3	81	73.6
pT4	4	3.6
N-stage		
pN0	32	29.1
pN1	78	70.9
Stage		
IA	3	2.7
IB	7	6.4
IIA	20	18.2
IIB	73	66.4
III	3	2.7
IV	4	3.6
Histological grade		
1	30	27.3
2	65	59.1
3	7	6.4
Perineural invasion		
Positive	36	34.6
Negative	68	65.4
PD-L1 in tumour cells^a^		
Positive	5	4.5
Negative	105	95.5
CD73+ tumour cell score^b^		
High	37	33.6
Low	73	66.4
CD73+ TILs^c^		
Positive	55	53.9
Negative	47	46.1
CD73+ stroma^d^		
Strong	38	34.5
Moderate	37	33.6
Weak	35	31.8
CD73+ vessels^e^		
High	68	61.8
Low	42	38.2

^a^Tumour samples were considered PD-L1 positive when > 1% of the tumour cells were positive for PD-L1

^b^The score (0–300) was formed by multiplying the proportion of stained tumour cells by the staining intensity (0–3). 90 was set as a cut off value

^c^Tumour samples were considered positive when >3% of lymphocytes were positive for CD73

^d^Tumour stroma positivity was considered weak, moderate or strong when < 5%, 5–16% or > 17% of the stromal area was stained, respectively

^e^Due to the strong staining intensity of vascular structures, 95% was set as a cut off value for CD73 positivity of vascular structures

### Associations between CD73 expression and other histopathological variables

We analyzed the associations between clinical and histopathological variables, cell-specific CD73 positivity and PD-L1 positivity in tumour cells **(**Tables 2 and 3**)**.

**Table 2:** Clinicopathological variables and their association with CD73 expression in tumour cells (CD73 + TC)

	CD73 + TC high *n* = 37		CD73 + TC low *n* = 73		*p* value
	*n*	%	*n*	%	
T-class					0.985
1	1	2.8	2	2.7	
2	7	19.4	15	20.5	
3	27	75.0	5	72.6	
4	1	2.8	3	4.1	
N-class					0.916
0	11	29.7	21	28.8	
1	26	70.3	52	71.2	
Grade					*0.013*
1	5	14.7	25	36.8	
2	24	70.6	41	60.3	
3	5	14.7	2	2.9	
Perineural invasion					*0.041*
Negative	7	20.6	29	40.8	
Positive	27	79.4	42	59.2	
CD73+ vessels					0.377
Negative	12	32.4	30	41.1	
Positive	25	67.6	43	58.9	
CD73+ TILs					0.930
Negative	15	45.5	32	46.4	
Positive	18	54.5	37	53.6	
CD73+ Stroma					0.328
Weak	9	24.3	26	35.6	
Moderate	12	32.4	25	34.2	
Strong	16	43.2	22	30.1	
PD-L1 TC					*0.025*
0	33	89.2	72	98.6	
1	4	10.8	1	1.4	
Immune cell score					0.947
Low	11	29.7	23	32.4	
Moderate	13	35.1	23	32.4	
High	13	35.1	25	35.2

**Table 3:** Interrelationship between clinicopathological variables and their association to 5-year DSS

Variables	T class	N class	Perineural invasion	Vessels CD73+	LC CD73+^a^	Stroma CD73+	TC CD73+^b^	PD-L1 TC +	ICS^c^	5-year DSS^d^
Grade	NS	NS	NS	0.073↑	NS	0.060↑	*0.013*↑	*0.010*↑	NS	*0.008*↓
T class		0.057↑	NS	NS	NS	NS	NS	*0.016*↓	*0.032*↓	NS
N class			NS	NS	*0.001*↑	NS	NS	NS	NS	NS
Perineural invasion				NS	NS	NS	*0.041*↑	NS	NS	NS
Vessels CD73+					*0.004*↑	*>0.001*↑	NS	0.072↑	0.056↑	NS
LC CD73+^a^						*0.023*↑	NS	0.059↑	*0.038*↑	NS
Stroma CD73+							NS	*0.007*↑	*0.030*↑	NS
TC CD73+^b^								*0.025*↑	NS	*0.021* ↓
PD-L1 TC +									NS	NS
ICS^c^										*0.014*↑

Nonsignificant (NS), *p* > 0.1; bold value, *p* < 0.05. Chi-square test. Arrows show the direction of association

^a^CD73+ Lymphocytes (low/high)

^b^CD73+ Tumour cells (low/high)

^c^Immune cell score

^d^Log rank test used

High-level CD73 expression in tumour cells (CD73^+^TC) was positively associated with PD-L1 expression, perineural invasion and histopathological grade (Table 2). CD73 positivity in TILs was significantly associated with lymph node metastasis. Lymphocytic CD73 positivity was also associated with staining positivity in both stroma and vascular structures (Table 3). In addition, CD73 positivity in vascular structures and stroma was associated with each other. There were no associations between CD73^+^TC and CD73 positivity in any other cell types in the tumour area.

PD-L1 positivity in tumour cells was also associated with CD73 staining positivity in stroma and also with high histopathological grade and low T class of the primary tumour.

### CD73 expression and survival

**Fig. 3:**
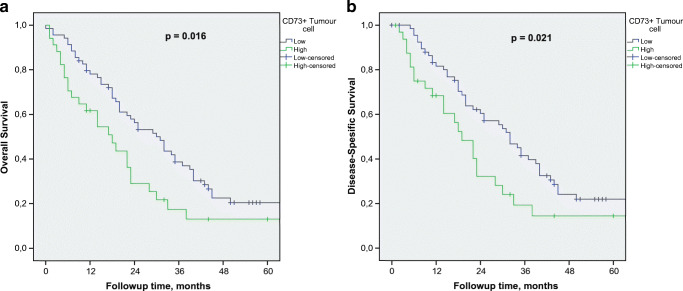
Prognostic impact of CD73^+^TC on DSS (a) and OS (b)

Regarding the whole study group, the median follow-up time was 44 (IQR 12.0 to 57.0) months for those alive at the end of follow-up. The estimated median overall survival (OS) for all patients was 23 [95% CI: (18.6–27.4)] months. CD73^+^TC was significantly associated with poor disease-specific survival (DSS) (*p* = 0.021) and OS (*p* = 0.016) (Fig. 3). In the multivariate analysis, CD73^+^TC was an independent negative prognostic factor together with histopathological grade, TNM stage and low ICS **(**Table 4**)**.

**Table 4:** Uni- and multivariate analysis with Cox proportional hazard model

	Univariate analysis (OS)^a^ HR (95% CI)	p	Univariate analysis (DSS)^b^ HR (95% CI)	*p*	Multivariate analysis (OS) HR (95%CI)	*p*	Multivariate analysis (DSS) HR (95% CI)	*p*
CD73 (TC)								
Negative	1	0.019	1	0.025	1	0.035	1	0.043
Positive	1.78 (1.10–2.88)		1.79 (1.08–2.97)		1.81 (1.04–3.15)		1.83 (1.02–3.29)	
Tumour grade								
1	1	0.006	1	0.012	1	0.006	1	0.011
2	1.96 (1.15–3.34)		1.96 (1.13–3.39)		1.96 (1.13–3.40)		2.00 (1.13–3.54)	
3	3.90 (1.55–9.85)		3.70 (1.37–10.04)		5.25 (1.69–16.30)		5.10 (1.48–17.61)	
TNM Stage								
IA	1.13 (0.27–4.64)	0.234	1.29 (0.31–5.32)	0.181	1.74 (0.40–7.66)	0.041	1.83 (0.41–8.14)	0.027
IB	0.54 (0.19–1.48)		0.57 (0.21–1.58)		0.33 (0.10–1.10)		0.36 (0.11–1.22)	
IIA	0.56 (0.29–1.07)		0.49 (0.24–1.00)		0.48 (0.25–.094)		0.40 (0.19–0.84)	
IIB	1		1		1		1	
ICS^a^								
High	1	0.019	1	0.018	1	0.004	1	0.003
Moderate	1.82 (1.03–3.19)		1.82 (1.01–3.30)		2.19 (1.17–4.13)		2.47 (1.27–4.82)	
Low	2.25 (1.26–4.05)		2.38 (1.29–4.40)		2.82 (1.51–5.23)		3.07 (1.59–5.95)	

^a^Immune cell score

High-level CD73 expression in tumour stroma, TILs or vascular structures did not show any significant correlation with survival (Table 3).

## Discussion

Our results show that a high CD73 expression in tumour cells is associated with poor survival in PDAC independently of ICS or TNM stage. We moreover found an association between high expression of CD73+ in tumour cells and perineural invasion. PD-L1 expression and high CD73 expression in both tumour cells and in stroma were significantly associated with each other. Moreover, we demonstrated that patients with high CD73 expression in TILs were more likely to have lymph node metastasis.

Earlier studies have reported similar results concerning the impact of CD73 on PDAC survival [28]. In a mouse experiment published by Stagg et al., CD73 deficiency led to increased number of CD8+ T cells in tumours. This was thought to be one factor behind the protective effect of CD73 deficiency [32]. According to another mouse model, high CD73 expression in T-lymphocytes was associated with an “exhausted” phenotype of T cells [33]. According to the present study, it is possible that, in PDAC, CD73 suppresses immune response by impacting on TILs activity rather than their number.

To the best of our knowledge, these results show for the first time the association between high CD73 expression in tumour cells and perineural invasion indicating that CD73 overexpression may be implicated in this process. According to the literature, perineural invasion can be found in some form in almost all surgically removed PDACs when searched with thin slice thickness and also taking account of perineural invasion with low severity. However, according to a meta-analysis of 3538 patients, the incidence of perineural invasion was 71.7%, which is in line with that found in our study population [34]. This discrepancy between the incidences found in routine histopathological analysis and in a meticulous search with thin slice thickness is thought to reflect the variable severity of perineural invasion. In other words, perineural invasion with low severity is sometimes not found in histopathological analysis when using routine slice thickness. The same meta-analysis, however, shows that perineural invasion found in routine histopathological analysis seems to be an independent prognostic factor for poor survival.

In our study cohort, high CD73 expression in both tumour cells and in stroma was significantly associated with PD-L1 expression in tumour cells. Similar findings have been reported in gastrointestinal neuroendocrine neoplasms [35]. Deng et al. demonstrated a close connection between these two immunosuppressive molecules in their recent mouse experiment concerning head and neck cancer [33]. They showed that blockade of CD73 reversed the exhausted T cell phenotype through downregulation of PD-1 and CTLA-4 on T cells. Mice studies have also proven that blocking adenosine receptor A2 (A2AR) enhances the efficacy of anti-PD-1 antibodies through enhanced antitumour T cell responses [36, 37].

Although the evidence of the pro-tumoural effect of high CD73 expression is increasing, the impact of CD73 expression in TILs is far from clear. Immunosuppressive regulatory T cells (T_reg_) as well as T helper 17 cells and myeloid-derived suppressor cells are known to express CD73 [38–40].We showed that PDAC patients with CD73 + TILs were more likely than the controls to develop lymph node metastases. We think this association may reflect the impact of immunosuppressive cells mentioned above. However, double staining of immune cells is needed in the future to confirm this hypothesis. Correspondingly, Ma et al. [41] showed that the increased expression of A2AR correlated with positive lymph node status in head and neck squamous cell carcinoma. This refers to the significance of the immunosuppressive adenosine pathway in cancer progression.

Our study has some limitations. Sampling error is a well-known risk related to the use of TMA. To minimize this risk, we analyzed whole sections of 16 cases to validate our method, and the correspondence between TMA and whole sections was excellent. The use of consecutive patient series from a single geographical area to avoid a selection bias strengthens our study. In addition, double assessing of IHC staining by two independent researchers increases the reliability of the results.

The development of the combined treatments of anti-CD73 with other immune-modulating agents such as anti-PD1 will potentially bring new hope for patients with PDAC. In the future, personalized cancer therapy will lead to an increasing need for applicable biomarkers. There also remains a need for basic research on our fine-tuned immune system.

In conclusion, our study shows that high expression of CD73 is an independent prognostic factor in PDAC also associated with perineural invasion. We furthermore demonstrate an association between CD73 and PD-L1 expression in pancreatic tumour cells. In addition, our study shows for the first time that patients with high CD73 expression in TILs are more likely to have lymph node metastasis.

## Abbreviations

PDAC: Pancreatic ductal adenocarcinoma
TIL: Tumour infiltrating lymphocyte
DSS: Disease-specific survival
OS: Overall survival
ICS: Immune cell score
